# A review of thalidomide and digestive system related diseases

**DOI:** 10.3389/fonc.2025.1543757

**Published:** 2025-05-29

**Authors:** Zhao Xi, Wang Jie, Zou Long, Shi Shasha

**Affiliations:** ^1^ The Third Hospital of Mianyang, Sichuan Mental Health Center, Mianyang, China; ^2^ Guangyuan Zhaohua District People’s Hospital, Guangyuan, China

**Keywords:** thalidomide, digestive diseases, bleeding, inflammation, tumors

## Abstract

Thalidomide, once discovered and used to treat pregnancy sickness, fell out of favor with teratogenicity scandals, and then re-entered the public eye with its amazing efficacy against leprosy and multiple myeloma, and its anti-tumor and anti-inflammatory effects were gradually discovered. In recent years, thalidomide has also begun to gain prominence for its role in digestive disorders, particularly for its anti-angiogenic effects, which are effective in the treatment of dysplastic gastrointestinal hemorrhage. The therapeutic effects of thalidomide in other digestive disorders, including radiation proctitis, inflammatory bowel disease, and malignant tumors of the digestive tract, such as liver, colorectal, gastric, esophageal, and pancreatic cancers, have been proposed in several clinical trials and case reports. Due to its potential adverse effects and controversial clinical utility, it has not been put into clinical use for digestive diseases in large numbers, but its unique therapeutic effects still warrant further study, so this paper analyses and summarizes the use, mechanism of action, and potential therapeutic uses of thalidomide in digestive diseases.

## 1 Introduction

Thalidomide was first synthesized in 1950 but was quickly withdrawn in 1962 due to reports of severe congenital abnormalities (1). Since 1965, thalidomide has been found to possess anti-inflammatory and anti-angiogenic properties and has been used under stringent conditions for the treatment of leprosy (2). In the 1980s and early 1990s, researchers discovered that thalidomide could treat certain autoimmune diseases (3, 4). In 1990, thalidomide was considered a first-in-class immunomodulatory drug (IMiD) due to its discovery in combination with other antineoplastic drugs for the treatment of multiple myeloma (1, 5), and was also considered a promising clinical ally for cancer immunotherapy (5). Thalidomide was approved by the US FDA for the treatment of Erythema nodosum leprosum (ENL) and myeloma in 1998 and 2006, respectively (6). A new generation of analogues of thalidomide was developed in the 2000s, including lenalidomide and pomalidomide, collectively referred to as immunomodulatory imide drugs (IMiD) because of their potent immunomodulatory effects (1, 7). Thalidomide has also appeared on the clinical scene more frequently due to the development of its analogues. In recent years, thalidomide has also been gradually found to be effective in many digestive disorders, including angiodysplastic gastrointestinal hemorrhage, radiation proctitis, inflammatory bowel disease, and gastrointestinal malignancies, such as hepatocellular carcinoma, colorectal carcinoma, gastric carcinoma, esophageal carcinoma, and pancreatic carcinoma. However, the current drug insert for thalidomide is only approved for tumor-type leprosy. Therefore the use of thalidomide in most diseases is not entered in the drug insert, such as it is an over-the-counter antiangiogenic therapy for patients with hereditary hemorrhagic telangiectasia, HHT (8), but thalidomide has been popularized in such patients, including epistaxis and visceral vascular malformation bleeding (9, 10) with excellent efficacy. Whereas thalidomide has been used in the treatment of malignant tumors, partly because of its direct antitumor effect and partly because of its ability to ameliorate the malignant state of the tumor.

There are many studies on thalidomide and its effects on digestive disorders, but it has not been put into clinical use for digestive disorders in large numbers due to its potential adverse effects and controversial clinical utility. However, its unique therapeutic effects still warrant further research. Therefore, this paper analyses and summarizes the use of thalidomide in digestive diseases, its mechanism of action and potential therapeutic uses, with a view to providing more strategic options for patient care.

## 2 Thalidomide mechanism

### 2.1 Chemical structure of thalidomide

The unpredictable toxicity of thalidomide is mainly due to its specific chirality, which is a racemic glutamate analogue consisting of S (–) and R(+) enantiomers, which are interconverted under physiological conditions and cannot be completely separated (11, 12). Although S (–) inhibits the release of tumor necrosis factor α and acts as an immunosuppressant, the presence of S (–) also contributes to the teratogenicity of thalidomide, whereas the R(+) form mainly acts as an antipregnant, antiemetic, and sedative (13).

### 2.2 Mechanism of action of thalidomide

Thalidomide exerts its therapeutic properties primarily through the modulation of cytokines, particularly interleukin-1 (IL-1), interleukin-6 (IL-6), interleukin-10 (IL-10), interleukin-12 (IL-12), interleukin-1β (IL-1β) and tumor necrosis factor-α (TNF-α). 12, IL-12), interleukin-1β (IL-1β) and tumor necrosis factor-α (TNF-α), interferon, cyclooxygenase 2, and nuclear factor κB (12). Thus, the main mechanisms of action of thalidomide include inhibition of angiogenesis, inhibition of cytokine-mediated pathways, inhibition of regulation of adhesion molecules, inhibition of cyclooxygenase-2 and inhibition of stimulation of immune responses. Its most prominent adverse drug reactions are drowsiness, constipation, dizziness and fatigue. As described in **Table 1**. The most important reason for limiting the use of thalidomide is its severe teratogenicity. Studies have indicated that thalidomide-binding protein (cereblon, CRBN) is the target of teratogenic effects, and that thalidomide exerts its teratogenic effects by binding to CRBN and inhibiting the inhibition of E3 ubiquitin ligase function (6). It has also been suggested that thalidomide-induced oxidative stress and antiangiogenic effects are possible and plausible reasons for the teratogenicity of thalidomide (14, 15).

**Table 1:** Thalidomide in digestive diseases.

Disease	Mechanism of action	Improvement	Adverse effects
Vascular dysplasia gastrointestinal hemorrhage	1. Inhibit angiogenesis: reduce abnormal blood vessel formation by down-regulating VEGF and β-FGF expression 2. Anti-inflammatory: inhibit TNF-α and NF-κB pathway, reduce vascular permeability	Significantly reduces the number of bleeds and the need for blood transfusions, with efficacy lasting part of the time after stopping the drug	Drowsiness, peripheral neuropathy, constipation, deep vein thrombosis (risk of long-term use)
Radiation enteritis	1. anti-inflammatory: inhibit neutrophil chemotaxis and inflammatory factor release 2. anti-angiogenic: reduce vascular proliferation after radiation injury	Delaying the onset of acute enteritis and reducing the severity of symptoms (e.g. bleeding, diarrhea)	Constipation, peripheral oedema, elevated liver enzymes
Inflammatory bowel disease	1. Immunomodulation: inhibit TNF-α, IL-12, regulate Th1/Th2 balance 2. Anti-angiogenesis: reduce neovascularisation at the site of inflammation	Relieve abdominal pain, diarrhea, promote mucosal repair, maintain remission period	Drowsiness, peripheral neuritis, constipation
Malignant tumors of the digestive tract	1. anti-angiogenesis: inhibit tumor blood vessel formation 2. Immunomodulation: enhance T-cell activity and inhibit tumor cell proliferation	Delaying tumor progression, reducing tumor cell migration	Deep vein thrombosis, peripheral neuropathy, somnolence (related to dose accumulation)
Diseases of the oral mucosa	1. Immunomodulation: inhibit TNF-α and IL-8, regulate the ratio of CD4+/CD8+ T-cells 2. Anti-inflammatory: reduce the release of inflammatory mediators	Shorten ulcer healing time and reduce recurrence frequency	Dry mouth, constipation, skin rash

## 3 Thalidomide and digestive disorders

### 3.1 Thalidomide for vascular dysplasia gastrointestinal hemorrhage

Gastrointestinal angiodysplasia (GIAD) can be found in any part of the gastrointestinal tract, and the most common cause of GI bleeding due to GI dysplasia is small bowel hemorrhage, however, small bowel hemorrhage has limitations in both endoscopic and surgical treatments, and ends up recurring leading to costly transfusion and treatment costs. As a result, thalidomide, a potent angiogenesis inhibitor, was discovered and Huimin Chen from Shanghai Jiaotong University found that thalidomide reduced the incidence of recurrent bleeding due to small bowel vascular dysplasia (16). After three months of treatment of patients with refractory severe recurrent intestinal bleeding, thalidomide strongly inhibited serum vascular endothelial growth factor levels compared with pre-treatment levels (17), thus concluding that thalidomide inhibits the growth of vascular endothelium, thus improving vascular dysplasia to achieve hemostasis. The ability of thalidomide to induce vascular maturation may also be a therapeutic strategy for the treatment of vascular dysplasia (16, 18). A review of capsule endoscopy after 3 months of thalidomide treatment showed a significant reduction in the number, size and color intensity of vascular dysplasia (19). Also, thalidomide is an effective and relatively safe treatment for patients with refractory bleeding due to vascular malformations of the gastrointestinal tract (20). Recent studies have found that thalidomide analogues have the same efficacy, and thalidomide analogues such as lenalidomide and pomalidomide have been found to be potentially effective in the treatment of HHT (9, 21) and, of course, visceral arteriovenous malformation (AVM) (9, 10). Currently available clinical studies confirm the major adverse events of thalidomide, specifically constipation, somnolence, limb numbness, peripheral oedema, dizziness and elevated liver enzyme levels (16). The main reason for discontinuation due to long-term use is because of neurotoxicity (22).

Thalidomide has also been reported in the treatment of recalcitrant bleeding due to portal hypertensive gastropathy (23). In addition recurrent bleeding from the watermelon stomach associated with cirrhosis cannot be controlled by argon knife coagulation therapy (APC), but can be successfully treated with thalidomide (24). Interestingly, GIAD is very common in patients with chronic renal failure (CRF) and its incidence increases further with the duration and severity of renal disease (25). Therefore thalidomide is also frequently used in patients with recurrent bleeding in renal failure.

### 3.2 Thalidomide for radiation enteritis

According to studies indicated that most cancer patients receive radiation therapy during the course of their disease, but radiation toxicity to normal tissues around the radiation range remains a major obstacle to disease control and quality of life in patients with localized tumors (26). Radiation enteritis is the most common complication of pelvic radiotherapy (27), and the treatment of radiation enteritis remains limited in the clinic, whether it is by medication, enemas, or the newest therapeutic modalities such as hyperbaric oxygen.

Pro-inflammatory factors such as TNF-α, IL-1 and IL-6 play an important role in the pathogenesis of the inflammatory response in acute radiation proctitis. Thalidomide is not only an angiogenesis inhibitor but also an anti-inflammatory agent (12). Studies have shown that thalidomide not only reduces the incidence and grade of acute radiation proctitis, but also improves the patient’s tolerated dose of radiotherapy and delays the onset of radiation proctitis, thus achieving effective prevention and treatment of acute radiation proctitis after postoperative intensity-modulated radiotherapy (IMRT) for cervical cancer (28).

### 3.3 Thalidomide for the treatment of inflammatory bowel disease

Thalidomide is used in the treatment of inflammatory bowel disease mainly because of its immunomodulatory effects. The potent immunomodulatory activity of thalidomide is mainly due to its ability to alter the secretion and activity of various cytokines, including IL-6, IL-10, IL-12, IL-1β, and TNF-α (29–31), suggesting that it may be effective against UC and CD.

TNF-α is a proinflammatory cytokine associated with CD (32), and thalidomide reduces the production of TNF-α (31). In 1999, thalidomide was shown to be effective in some patients with refractory Crohn’s disease. In this study, 22 patients with refractory Crohn’s disease were initiated on treatment with thalidomide, and 16 patients completed 4 weeks of treatment (12 clinical responses, 4 clinical remission), and 14 patients who completed 12 weeks of treatment met clinical response criteria (33). And low-dose thalidomide (50 mg) also appeared to be well-tolerated and effective at 12 weeks (34). thalidomide has also been shown to improve clinical remission rates in refractory Crohn’s disease in children and adolescents in a randomized controlled study in children and adolescents by Italian investigators in 2013 (35). Thalidomide treatment is equally effective in most patients with refractory active intestinal and/or perineal CD (36). In thalidomide in the treatment of refractory ulcerative colitis, complete clinical remission was achieved in 78.6% of patients treated with a 50 mg dose for 8 weeks (37). However, a high-quality randomized controlled study in 2015 concluded that thalidomide was only effective in inducing remission of CD in children, and current evidence is insufficient to support the use of thalidomide to induce remission of UC or adult CD (38). Thus the role of thalidomide in inflammatory bowel disease remains controversial, and the most recent study in 2022 by Peng Xiang et al. concluded that 100 mg of thalidomide taken continuously for 8 weeks can be used for induction and maintenance therapy of refractory CD in adults (39). However, its toxicity limits its use as maintenance therapy, and neuropathy is the main reason for discontinuation of long-term thalidomide use (35, 36). Interestingly, the powerful XGBoost algorithm of Chinese researchers accurately predicted thalidomide-induced peripheral neuropathy (TiPN) using 18 clinical features and 14 genetic variables. Its ability to identify high-risk patients using single nucleotide polymorphisms provides a viable option for improving thalidomide efficacy in patients with CD (40), but this algorithm is not currently in clinical use.

### 3.4 Thalidomide in the treatment of primary biliary cirrhosis

Primary biliary cirrhosis (PBC) is a chronic progressive cholestatic disease and an autoimmune disorder. A double-blind randomized controlled trial of thalidomide for the treatment of PBC was reported only in 1994 by the British physician P A McCormick (41), which indicated that apart from ursodeoxycholic acid, which is the only approved drug, drugs such as thalidomide, methotrexate, penciclovir, or colchicine are unlikely to be effective in altering the natural course of primary biliary cirrhosis (41, 42).

### 3.5 Thalidomide for the treatment of digestive malignancies

Thalidomide is currently used as an antineoplastic therapeutic agent in multiple myeloma (43–46), melanoma (47), glioblastoma multiforme (48), and renal cell carcinoma (49, 50). Among malignant tumors of the digestive system, only studies have shown some anti-tumor effects on hepatocellular liver cancer and colorectal cancer (51–55). Among liver malignancies, antitumor effects have only been found against cirrhosis-associated hepatocellular carcinoma (HCC), probably because the rich vascular distribution of HCC provides an attractive target for anti-angiogenic therapy that may be tolerated by cirrhotic patients, and a phase II clinical study in 2005 indicated that thalidomide may render the stabilization in patients with HCC (51). Longer patient survival may be achieved with thalidomide in combination with other chemotherapeutic agents at different doses, or with thalidomide analogues (51). In colon cancer, studies have indicated that thalidomide is an effective adjuvant antineoplastic agent, and in a clinical study of 60 colon cancer cases, it was concluded that combining thalidomide had a significant therapeutic effect in high-risk patients undergoing surgery for stage II and III colon or rectal cancer who received downstaged, first-line, palliative oxaliplatin + capecitabine chemotherapy after surgical resection of metastatic colon cancer (56). Radioimmunotherapy (RIT) in combination with thalidomide antiangiogenic therapy produces a better tumor response than monotherapy, and antiangiogenic therapy may prolong the dormancy of cancer lesions (55). Irinotecan is the only accepted second-line treatment for colorectal cancer in the United States (57), however dosing is often limited by associated late-onset diarrhea, and studies have found that thalidomide virtually eliminates the dose-limiting gastrointestinal toxic effects of irinotecan, particularly diarrhea and nausea (58).

As for solid tumors of the digestive system other than hepatocellular carcinoma and colorectal cancer, thalidomide has been found to improve malignant tumor cachexia (59). A large number of patients with advanced cancer develop malignancy before death, and it is more common in malignant tumors of the digestive system, including pancreatic, gastric, esophageal, and colorectal cancers. However, there are no effective drugs for treating cancer cachexia, and promising pharmacological treatments are limited, including megestrol acetate, anamorelin, thalidomide, and delta-9-tetrahydrocannabinol (60). In contrast, thalidomide alters the cytokine triggers of the wasting response through its potent anti-TNF-alpha action, inhibiting the production of transcription factor (NkB), which limits downstream gene expression, which in turn affects the control of pro-inflammatory cytokines, cellular growth, and regulation, ultimately leading to an amelioration of the weight loss associated with malignant stroma (61).Gordon JN also found that thalidomide was effective in attenuating weight loss in patients with advanced weight and lean body mass loss in patients with pancreatic cancer cachexia (59). And Khan ZH’s study concluded that thalidomide also reversed weight loss in patients with advanced esophageal cancer within 2 weeks (62). However, a 2012 study analyzing large amounts of data ultimately did not provide a positive answer and there was insufficient evidence to recommend it for clinical practice (61).

### 3.6 Thalidomide for the treatment of oral mucosal diseases

Thalidomide has been widely used in oral mucosal diseases, such as oral lichen planus, recurrent aphthous ulcers, leucosis involving the oral mucosa, oral cancer, etc (63–65).

National guidelines for the treatment of oral lichen planus recommend a starting dose of 50–100 mg/d of thalidomide, which is gradually reduced to the lowest effective dose (65, 66). Thalidomide has shown some efficacy in controlling the frequency of recurrence of recurrent aphthous ulcers, and should be used mainly in the heavy or recalcitrant type, but national and international guidelines do not explicitly recommend the dose to be given (67, 68). To reduce adverse effects, some studies have used reduced dosing regimens, and although they have been shown to significantly prolong the ulcer interval in patients, none have compared the efficacy and safety of different doses of thalidomide (69, 70).

There is increasing evidence from *in vitro* and *in vivo* experiments that thalidomide is a promising anticancer agent for oral cancer, and therefore there is a strong need for more clinical trials with larger sample sizes to demonstrate the potential role of thalidomide in the routine clinical management of oral disease (63). With the expansion of thalidomide’s clinical scope of application, the adverse drug reaction (ADR) caused by thalidomide has been gradually taken into account, especially the damage to the nervous system, and the potential teratogenicity of thalidomide should be highly concerned for the population of childbearing age (71, 72).

Therefore, more high-quality clinical trials of thalidomide for the treatment of oral mucosal diseases are needed to determine the optimal dosing regimen and safe dosage to enhance the safety of the drug for patients.

## 4 Conclusions and outlook

Thalidomide was once restricted because of its teratogenicity, but as more and more of its effects continue to be discovered, it is now well used in clinical treatment. For example, multiple myeloma and some skin diseases. However, at present, the use of thalidomide in digestive system diseases is not popular, but its over-the-counter use in digestive system diseases is not uncommon. This includes the treatment of unexplained gastrointestinal hemorrhage, which is first considered to be due to vascular dysplasia and congenital capillary malformations. While its use in inflammatory bowel disease has been found to induce remission of CD and UC, a high-quality RCT conducted by Canadian pediatrician C Yang concluded that thalidomide was only effective in inducing remission of CD in children (38). The use of thalidomide for the treatment of radiation proctitis is similarly controversial, but there are fewer clinical studies in this area, and correspondingly fewer reports, but because of its controversial clinical role and greater susceptibility to adverse effects, it has not been put into clinical use in large numbers.

At present, the higher adverse reaction rate of thalidomide remains a pressing issue, but its utility in improving the malignant status of tumors remains promising, and its clinical application, including gastrointestinal tumors, still requires extensive clinical research and practice.
